# Liver Stiffness Measurement and Biochemical Markers in Senegalese Chronic Hepatitis B Patients with Normal ALT and High Viral Load

**DOI:** 10.1371/journal.pone.0022291

**Published:** 2011-07-25

**Authors:** Papa Saliou Mbaye, Anna Sarr, Jean-Marie Sire, Marie-Louise Evra, Adama Ba, Jean Daveiga, Aboubakry Diallo, Fatou Fall, Loic Chartier, François Simon, Muriel Vray

**Affiliations:** 1 Department of Hepatology and Gastroenterology, Principal Hospital, Dakar, Senegal; 2 Department of Hepatology and Gastroenterology, Abass Ndao Hospital, Dakar, Senegal; 3 INSERM U941, University of Medicine Paris-Diderot, Saint-Louis Hospital, Paris, France; 4 Medical Laboratory, Pasteur Institute, Dakar, Senegal; 5 Department of Hepatology and Gastroenterology, Saint-Jean de Dieu Hospital, Thies, Senegal; 6 Department of Hepatology and Gastroenterology, Grand-Yoff Hospital, Dakar, Senegal; 7 Epidemiology Unit of Infectious Diseases, Pasteur Institute, INSERM, Paris, France; Yonsei University, Republic of Korea

## Abstract

**Background and Aims:**

Despite the high prevalence of chronic hepatitis B (CHB) in Africa, few studies have been performed among African patients. We sought to evaluate liver stiffness measurement by FibroScan® (LSM) and two biochemical scores (FibroTest®, Fibrometer®) to diagnose liver fibrosis in Senegalese CHB patients with HBV plasma DNA load ≥3.2 log_10_ IU/mL and normal alanine aminotransferase (ALT) values.

**Methods:**

LSM and liver fibrosis biochemical markers were performed on 225 consecutive HBV infected Senegalese patients with high viral load. Patients with an LSM range between 7 and 13 kPa underwent liver biopsy (LB). Two experienced liver pathologists performed histological grading using Metavir and Ishak scoring.

**Results:**

225 patients were evaluated (84% male) and LB was performed in 69 patients, showing F2 and F3 fibrosis in 17% and 10% respectively. In these patients with a 7–13 kPa range of LSM, accuracy for diagnosis of significant fibrosis according to LB was unsatisfactory for all non-invasive markers with AUROCs below 0.70. For patients with LSM values below 7 kPa, FibroTest® (FT), and Fibrometer® (FM) using the cut-offs recommended by the test promoters suggested a fibrosis in 18% of cases for FT (8% severe fibrosis) and 8% for FM. For patients with LSM values greater than 13 kPa, FT, FM suggested a possible fibrosis in 73% and 70%, respectively.

**Conclusion:**

In highly replicative HBV-infected African patients with normal ALT and LSM value below 13 kPa, FibroScan®, FibroTest® or Fibrometer® were unsuitable to predict the histological liver status of fibrosis.

## Introduction

More than 350 million patients worldwide infected with hepatitis B virus (HBV) are living with chronic hepatitis B (CHB) [1]. Senegal ranks among the countries with the highest prevalence in the world; 17% of blood donors test positive in plasma for hepatitis B virus surface antigen (HBsAg). Primarily infected during early childhood, this population shows a high rate of precore mutation, around 90% (HBeAg-negative) [2]. Without treatment, 15 to 40% of subjects with chronic HBV infection will develop cirrhosis and face a risk of developing hepato-cellular carcinoma [3]. European updated guidelines for chronic hepatitis B recommended assessing liver fibrosis in patients with HBV plasma DNA load above 2,000 IU/mL or elevated ALT [4]. American guidelines differ concerning HBeAg status; they recommend liver biopsy (LB) for HBeAg-negative patients with persistent HBV DNA above 2,000 IU/mL and ALT level ≤2 ULN [5].

Treatment is recommended when LB shows moderate/severe necroinflammation or significant fibrosis by METAVIR scoring [4]–[5]. Nevertheless, LB is an invasive procedure and has rare but potentially life-threatening complications [6]–[7]. Also, despite being considered the “gold standard” there can be marked inter- and intra-observer variability leading to incorrect staging in up to 33% of biopsies [8]–[10]. Moreover, it is difficult to perform in developing countries because of its cost and the limited number of histopathologists.

In resource-poor contexts, surrogate markers that enable the non invasive measurement of fibrosis in CHB patients and serve as an alternative to liver biopsy are badly needed. These markers include a physical device that measures liver stiffness by elastometry (FibroScan®) and biochemical scores developed in industrialized countries. FibroScan®, FibroTest®, Fibrometer®, APRI, Hepascore and Fib-4 have been evaluated in Caucasian populations with chronic hepatitis C (CHC) and, show good correlation with liver fibrosis stage [11]–[17]. Strategies combining biochemical fibrosis scores or one biochemical score with liver stiffness measurement (LSM) by FibroScan® have decreased the need for LB in patients with viral hepatitis; [18]–[20] such strategies currently have already been widely implemented for hepatitis C patients, particularly in France [21].

The picture is not so clear with CHB [21]–[27]. Whilst studies have shown a correlation between hepatitis B virus (HBV) viral load and liver fibrosis in HBeAg negative patients [28] there is less evidence of a correlation between biochemical scores and liver stiffness measurement for CHB, particularly in countries with high CHB prevalence, with only one study involving CHB African patients [29]. We conducted a study of HBV-infected Senegalese patients with normal ALT values but elevated HBV DNA loads with the following objectives:

1- To compare the results of LSM and biochemical scores (FibroTest®, Fibrometer®).
2- To compare liver biopsy with LSM and biochemical scores (FibroTest®, Fibrometer®) for predicting liver fibrosis in patients with LSM between 7 and 13 kPa.

## Materials and Methods

Patients were consecutively enrolled by private practitioners and by four public hospitals in Dakar, Senegal's capital city, from December 2006 to June 2008. Treatment-naïve patients above 18 years old, with positive HBsAg over six months, symptom-free, HIV, HCV and HDV negative, and with a serum HBV DNA level ≥3.2 log_10_ IU/mL were eligible for enrolment. All study participants underwent LSM, FT and FM on the same day. The study's scientific committee limited the LB to patients with LSM values ranging between 7 kPa and 13 kPa. This decision was based on published studies of CHC patients, in which patients with LSM≤7 kPa were assumed to have ≤F1 METAVIR stage. Conversely, people with LSM≥13 kPa were assumed to have F4 METAVIR stage, and thus no indication of LB [30].

The protocol was in accordance with Declaration of Helsinki ethical guidelines and was approved by the Senegalese ethics committee. Patients fulfilling the inclusion criteria were enrolled after providing written and informed consent. Patients eligible for treatment were prescribed Lamivudine free of charge and included in a national hepatitis B program.

### Liver stiffness measurements (LSM) of FibroScan®

LSM was performed in the right lobe of the liver through the intercostal spaces, with the patient lying in the dorsal decubitus position, right arm in maximal abduction. After receiving expert training, four physicians performed LSM. Several successful acquisitions were performed on each patient. The result was expressed as the median value of 10 successful acquisitions. The inter-quartile range (IQR) was also assessed.

### Liver histology and fibrosis quantification

LB was performed within six months of non-invasive markers evaluation.

Liver biopsies were obtained using 16G disposable needles (Hepafix; B. Braun, Melsungen, Germany). Fibrosis staging was considered reliable when the liver specimen length was ≥15 mm or the portal tract number ≥10 [31].

Liver specimens were stained with hematoxylin-phloxin-saffran and picrosirius red and interpreted by two highly experienced liver pathologists (MC, JLS), who were unaware of LSM, clinical, and biological data.

Liver fibrosis was scored on a 0–4 scale according to the METAVIR scoring system [32]. Necroinflammatory activity, based on assessment of interface activity and lobular necrosis, was graded on a 4-point scale [32].

### Serum markers of fibrosis

Two non invasive serum methods were assessed: FibroTest® [12], and Fibrometer® [13]. The following parameters were determined on blood sampled the same day as LSM onto a Vitros automat (Ortho Clinical Diagnostics, Issy-les-Moulineaux, France): aspartate aminotransferase (AST), alanine aminotransferase (ALT), γ-glutamyl transpeptidase, total bilirubin, platelet count, HBe antigen, urea and prothrombin time.

Alpha2-macroglobulin, apolipoprotein A1, haptoglobin, hyaluronic acid and hepatitis B viral load were performed in a specialized laboratory (Biomnis, Lyon, France). Scores for the FibroTest® (FT) were calculated by Biopredictive (Paris, France). Biolivescale (Pr Calès, Angers, France) generously provided scores for the Fibrometer® (FM). All patients in whom haemolysis could influence the biochemical scores were excluded from the analysis.

Both FT and FM markers were evaluated blindly to the results of LSM and LB. Results were evaluated in both quantitative and METAVIR scores from F0 to F4.

### Virological analyses

The HBV DNA quantification was assayed using the Cobas AmpliPrep/Cobas Taqman HBV test, v1.0 assay (Roche Diagnostics, Meylan, France), with a detection threshold of 12 IU/mL (1.1 log_10_ IU/mL).

### Statistical analysis

Quantitative variables were expressed as mean±standard-deviation (SD) or median and interquartile range [IQ1–IQ3] and discrete variables by percentages. Differences among percentages were analyzed using the Fisher's exact test.

LSM was categorized in three classes: <7 kPa, between 7 and 13 kPa, and ≥13 kPa. The number of patients with an IQR>33% of the results of the examination was given.

Agreement between the two pathologists for METAVIR of liver specimens was evaluated using the Kappa coefficient with the interpretation scale of Landis-Koch [33].

A comparison of METAVIR staging from LB to LSM and biochemical markers was conducted only on patients with LSM values between 7 and 13 kPa.

With LB as the gold standard, the diagnostic performance of each non-invasive marker was evaluated by performing the Area under the ROC curve (AUROC) with 95% confidence interval (CI). AUROCs were compared with the rocgold procedure [34].

Statistical analysis was performed using the STATA 10 software.

## Results

### Patients

Eight hundred seventy four consecutive patients who were HBsAg positive for over six months were screened by seven hepatologists in Dakar. Eighty-three patients with ALT values above the normal were ineligible based on study criteria.

**Figure 1:**
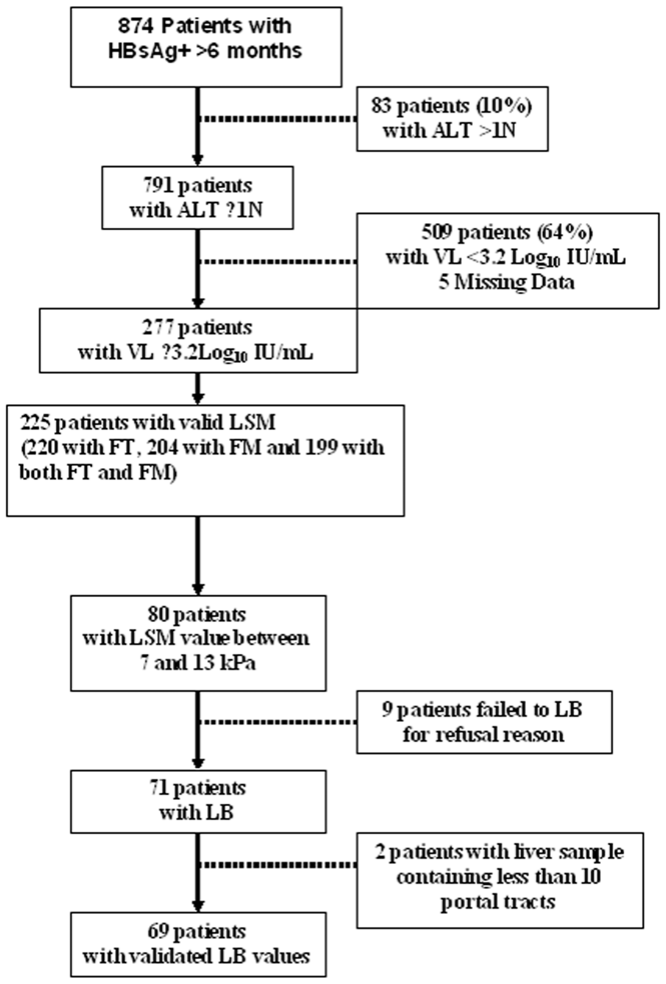
Flow chart.

Among the 791 with normal ALT values, 277 patients had HBV DNA level ≥3.2 log_10_ IU/mL and 226 underwent LSM. Only one patient failed to undergo LSM because of fatty thorax (Figure 1).

### Patient characteristics

The population was primarily male (84%) with a mean age of 30 years. The median viral load was 3.6 log_10_ IU/mL, and 91% of patients were HBeAg negative. LSM ranged from 3.3 to 39.1 kPa, with median value of 6.6 kPa. Twenty seven patients (12%) had IQR values greater than 33%. One hundred and thirty three patients (59%) had values of less than 7 kPa, 80 (36%) had values between 7 and 13 kPa, and 12 (5%) had values greater than 13 kPa (Table 1).

**Table 1:** Main characteristics of the 225 patients with CHB.

**Demographics**	
Sex, Male (n,%)	188 (84)
Age* (yrs)	30±8
BMI* (kg/m2)	21.4±3.3
**HBV infection**	
HBe antigen positive (n,%)	21 (9)
HBV viral load* (Log IU/mL)	4.0±1.1
≥4.2 Log IU/ml (n,%)	50 (22)
**Biochemical data**	
Platelets* (10^3^/mm^3^)	195±53
Prothrombin time* (% of normal)	85±6
<80 (n,%)	66 (29)
Total bilirubin* (mol/L)	13.4±6.0
γglutamyl transpeptidase* (IU/L)	31±24
AST* (IU/L)	35±22
ALT* (IU/L)	36±10
**Non Invasive Fibrosis Markers**	
**LSM values** ** **(kPa)**	**6.6 (5.4–8.7)**
IQR>33% (n,%)	27 (12)
<7 (n,%)	133 (59)
[7–13[ (n,%)	80 (36)
≥13 (n,%)	12 (5)
**FibroTest®** *	**0.32 (0.21–0.49)**
<F2 (n,%)	107 (49)
≥F2*** (n,%)	113 (51)
**Fibrometer®** *	**0.45 (0.34–0.58)**
<F2 (n,%)	64 (31)
≥F2** *(n,%)	140(69)

*mean±SD,

**Median [IQ1–IQ3],

***F1–F2 considered as ≥F2.

Fifty-one percent of the patients scored ≥2 (with F1–F2 considered to be F2) for the FT, while 69 percent of the patients scored ≥F2 for the FM. Using this threshold of F2 to initiate treatment, the serum markers were in concordance in 139 patients (70%) and differed in 60 patients (30%).

The proportion of patients with values equal to F1–F2, i.e. in the grey zone, was 26% (58/220) and 50% (102/204) for FT and FM markers, respectively.

The proportion of patients identified as F3–F4 was 18% (40/220: 26 F3, 2 F3–F4 and 12 F4) for FT and 2.5% (5/204) for FM markers.

### Comparison between histology and non invasive markers

A liver biopsy was performed in 71 of the 80 patients with LSM values between 7 and 13 kPa. The other nine patients declined to be biopsied. For two patients, the LB result was not retained because the LB specimens contained less than 10 portal tracts. Therefore, comparison between LB and LSM, FT and FM could be made in 69 patients.

For 64 patients (93%), the period between liver biopsy and non-invasive markers evaluation was less than two months, with all patients biopsied within a delay of less than 6 months.

The median biopsy length was 30 mm [25]–[34], with a median of 24 [20]–[30] portal tracts.

Fifty (72%) of the 69 patients had absent/mild fibrosis (METAVIR F0–F1), 12 (17%) had significant fibrosis (F2) and 7 (10%) had severe fibrosis (F3). There was no report of F4 stage.

In patients identified with a fibrosis stage equal to F2 at LB, LSM ranged from 7.1 to 11 kPa. In patients with a fibrosis stage equal to F3, LSM values ranged from 7.8 to 12 kPa.

The grades of activity were classified as A0 in 43 cases (62%), A1 in 20 cases (29%) and A2 in six cases (9%).

The Kappa coefficient between the two pathologists for significant fibrosis was 0.71±0.11.

**Figure 2:**
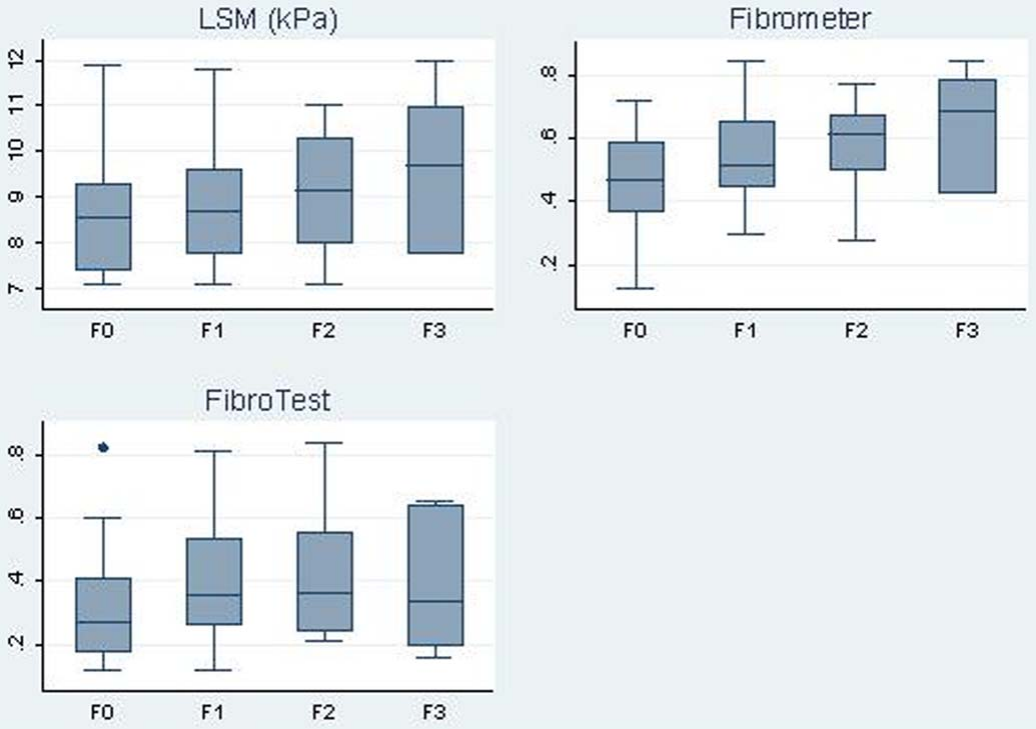
Box plots of LSM, FibroTest® and Fibrometer® scores according METAVIR stages from Liver Biopsy.

Figure 2 shows box-plots of LSM and the two biochemical scores versus METAVIR fibrosis stages. There was no clear correlation between the values of the LSM and two biological markers and the LB METAVIR Scores.

The AUROC values for FibroScan®, FibroTest® and Fibrometer® were 0.61 [CI95%: 0.45–0.77], 0.55 [CI95%: 0.39–0.71], and 0.68 [CI95%: 0.53–0.82], respectively. No differences were observed when patients with an IQR for LSM greater than 33% were excluded from the analysis.

Comparison of AUROCs of all non-invasive markers two by two detected no significant difference.

When considering the 12 patients with LSM values greater than 13 as having a fibrosis stage equal or greater than F2, the performances of the three non-invasive markers were improved, especially for LSM with an AUROC value of 0.76 [0;64–0.88].

### Comparison of FibroTest® and Fibrometer® results expressed as METAVIR scoring stages and LSM values

Table 2 reports results of FT and FM expressed as METAVIR-like scores and LSM values.

**Figure 3:**
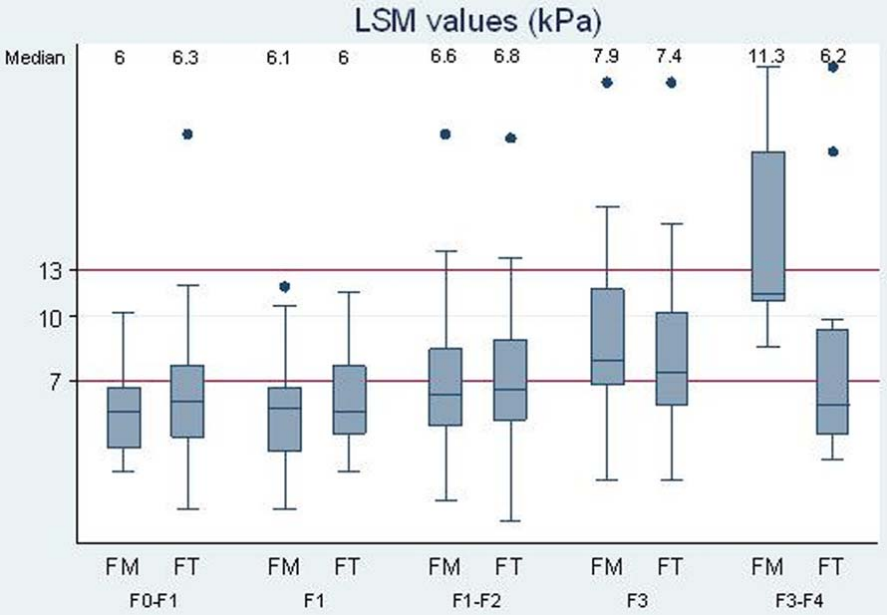
LSM values for each FibroTest® and Fibrometer® stage of fibrosis.

For patients with LSM<7 kPa, 43% of patients were classified as F1–F2 or ≥F2 by FT and 57% by FM. Among the 116 patients with both FT and FM results, 39 patients (34%) were classified as F1–F2 or with stages requiring a treatment (≥F2). No patients had Metavir stage ≥F3 by FM. Conversely, seven patients, all men, displayed a Metavir score ≥F3 (2 patients F3–F4 and 5 patients F4) by FT. Table 3 summarizes for these seven patients the parameters used to calculate FT scores and FM scores. For six out of seven subjects, haptoglobin value was very low (<0.1 g/L). Haemolysis was not reported for these patients. In contrast, among the 121 patients with LSM<7 kPa and Metavir stages by FT<F3, only six (5%) had haptoglobin value <0.1 g/L.
For patients with LSM values over 13 kPa, 73% and 70% of patients had an FT and FM equivalent of ≥F2–F3, respectively, with one patient having a FT measure of ≤F1 (F1–F2 by FM), one patient measuring F1–F2 by both serum markers, and one patient measuring F3 by FT but F1–F2 by FM.
Among the 73 patients with LSM values between 7 and 13 kPa, nine (12%) were classified ≤F1 by the two markers, and 40 (55%) were classified ≥F1–F2 for both markers (figure 3).

**Table 2:** Results of FT, FM METAVIR scoring stages according to Liver Stiffness Measurement values.

LSM (kPa)	≤F1	F1–F2	F2–F3	F3–F4	Total
	n (%)	n (%)	n (%)	n (%)	n
<7					
FT	74 (57)	32 (25)	15 (12)	8 (6)	129
FM	52 (43)	59 (49)	9 (8)	0 (0)	120
7–13					
FT	32 (40)	24 (30)	20 (25)	4 (5)	80
FM	12 (16)	40 (55)	18 (25)	3 (4)	73
≥13					
FT	1 (9)	2 (18)	6 (55)	2 (18)	11
FM	0 (0)	3 (30)	5 (50)	2 (20)	10

**Table 3:** Values of parameters used to scoring FibroTest® and Fibrometer® in the 7 patients with LSM<7 kPa and FibroTest® METAVIR scoring stages ≥F3.

Age	PT	TB	GGT	Platelets	HA	Urea	A2 M	Hapto	ApoA1	ALT	AST	LSM	FT	FM
19	99	22.2	27	42	25	2.82	3.81	.78	.59	39	31	4.8	F4	F2–F3
25	94	22.2	33	214	19	5.98	2.94	<0.1	1.62	52	37	5.3	F3–F4	F1–F2
30	93	12	30	169	19	4.48	2.89	<0.1	1.2	38	38	5.6	F3–F4	F1–F2
23	76	8.6	21	157	19	2.99	4.6	<0.1	.90	31	35	4.6	F4	F2–F3
24	56	25.7	24	167	22	3.65	3.37	<0.1	1.2	35	53	6.3	F4	F2–F3
26	84	12	45	237	19	1.99	3.29	<0.1	1.11	40	30	6.1	F4	F1–F2
24	80	12	31	196	58	4.32	3.1	<0.1	1.12	31	26	5.3	F4	F1–F2

Normal values: PT (prothrombin time):>70%, TB (total bilirubin): <20 µmol/L, GGT<73 IU/L, Platelets:>150 Giga/L, HA (Hyaluronic acid) <100 µg/L, Urea: 3.3–8.3 mmol/L, A2M (Alpha-2 macroglobulin): 1.3–3 g/L, Hapto (Haptoglobin): 0.64–1.7 g/L, ApoA1 ( Apolipoprotein A1): 1.04–2.02 g/L, ALT: 72 IU/L, AST: 59 IU/L.

Conversely for patients classified as F3–F4 for FT or FM, the median LSM value was 8 and 10.5 kPa, respectively. For patients with FT or FM Metavir stages ≤F1–F2, LSM values were less than 13 kPa; the median LSM was less than 7 kPa. No clear relationship was identified between LSM values and FT or FM scores.

## Discussion

In the present study conducted on 874 patients with HBV infection, only 10% displayed an ALT value exceeding the normal level. For most HBV-infected patients with normal ALT, monitoring remains problematic. Liver enzymes are generally the only markers routinely available in developing countries, and thus patients with normal ALT go untreated.

In Senegal, 17% of the population is considered to be HBV infected, and more than 60% of all children are infected with HBV by five years old [35]. The high prevalence of mutant precore in this Senegalese population could be explained by the virus's long evolution and selective strain bottleneck. This high prevalence of mutant precore limits the use of HBe antigen detection as a surrogate marker of HBV replication [2]. Since techniques of measuring viral load for HIV monitoring are available in many African countries, they could also be used in HBV quantification.

Patients with significant viral loads should undergo a liver fibrosis assessment before considering antiviral treatment. In this study among the patients with normal ALT values, 36% had a viral load greater than 3.2 log_10_ IU/mL, and 22% had values greater than 4.2 log_10_ IU/mL, reflecting an active infection, as LB results confirmed. Liver biopsy, however, remains difficult to perform in resource-poor countries, and non invasive markers are thus crucial to identifying patients who need treatment. The FibroScan® could be made more readily available, because the device is easy to use and the equipment simple to maintain. In contrast, expensive biochemical tests like FibroTest® or Fibrometer® are less likely to be used, since most patients cannot afford them and local laboratories often cannot perform such specialized tests.

Among the 69 patients with LSM between 7 and 13 kPa and an LB, 27% had significant fibrosis and therefore should have received treatment despite normal ALT. We found no cases at the cirrhosis stage. The quality of the biopsy was high because of large specimen sizes; the Kappa coefficient confirmed the consistent agreement between the two pathologists. The accuracy of the non invasive markers was rather low, with values of AUROC less than 0.70 for all three markers.

Various studies have already been performed using these markers in hepatitis C infected patients, with good results in diagnosing significant fibrosis and an AUROC of more than 0.80 in most studies [17], [19], [36]. Nevertheless, accuracy is always lower in distinguishing absent/mild fibrosis (F0 or F1) from moderate fibrosis to cirrhosis (F2, F3, F4). For CHB patients, previous studies have been published on non-invasive markers, including one in Africa [29]. Leroy et al. compared patients with CHB versus those with hepatitis C for the performance of several non-invasive markers, including FT and FM, and recorded poorer results in CHB patients in diagnosing early stages of fibrosis [17]. Marcellin et al. showed that the performance of LSM in predicting liver fibrosis in patients with CHB is comparable to that observed in CHC patients. However, cut-off values differed slightly [23]. One explanation could be that nodular fibrosis in CHB patients was less extensive than that observed in CHC patients at the same METAVIR stage. Sebastiani et al. showed a poorer performance of non invasive markers in CHC patients with normal transaminases [37].

A recent study conducted in another west African country, Burkina Faso, reported better results for FibroTest®, Fibrometer® and FibroScan® markers to diagnose significant fibrosis with AUROCs of approximately 0.80 for Fibrometer® and FibroTest® and 0.87 for the FibroScan® [29].

One explanation of the discrepancy between our results and those from the Burkina study may be the larger proportion of significant fibrosis among the Burkinabe population due to the difference in the inclusion criteria (70% of patients presented with a METAVIR stage at least equal to F2). The heterogeneous recruitment of the Burkinabe study, which included patients already receiving antiviral treatment, could account for the better results.

Conversely, patients selected in our study represent a very homogeneous population of chronic HBV patients, naïve to treatment and newly referred, but the proportion of patients with significant or severe fibrosis (12 with F2, 7 with F3, and none with F4) do not allow any firm statistical conclusion on the correlation between liver histology and non invasive markers.

In our study, we focused on FT and FM, as these biochemical markers are validated and widely implemented for HCV, in France. Low-cost, easier and simpler non-invasive methods of assessing liver fibrosis such as APRI or Hepascore showed similar poor performances with AUROCs at 0.62, CI95%: [0.45–0.79] and 0.63, CI95%: [0.47–0.78] respectively (data not shown).

With regard to the three non-invasive markers performed on the entire population of 225 patients, a large proportion of subjects displayed values that fell into the grey zone F1/F2: 36% ranged between 7 and 13 kPa for FibroScan®, 26% for Fibrometer® and up to 51% for FibroTest®.

For subjects with an LSM value of less than 7 kPa and for whom treatment is not in theory indicated, we found 6% of subjects for whom the FT indicated a severe fibrosis; 12% of patients tested with the FT and 8% with FM revealed significant fibrosis. Haptoglobin values could explain the major disparity observed between LSM and FT. Dakar is now malaria-free, but chronic malaria and sickle-cell anaemia which may alter liver function were not examined in this study.

If we apply the cut-off of 7.2 kPa (close to 7) for LSM (defined in Marcellin et al.'s recent study of 200 CHB patients as the threshold for treatment), the rate of discordance among the three markers is high. We reached the same conclusions when we used the optimal cut-off of 7.3 kPa, the identical value found by Bonnard in Burkina Faso to identify patients with significant fibrosis.

For subjects with values between 7 and 13 kPa, results were more consistent, since all stages of fibrosis from F0 to F3 were observed; nonetheless, discordance with LB was significant. In contrast, all 11 subjects except two with a LSM value greater than 13 kPa had at least one of the two serum markers indicating significant or severe fibrosis (9/11); in eight out of 11 cases (73%), both FT and FM were concordant and thus indicated significant or severe fibrosis.

The selection of our population for biopsy based on intermediate values of LSM (7–13) skewed our METAVIR fibrosis staging on F1 and F2, the most difficult stages to differentiate. Furthermore, liver biopsy itself has an intrinsic variability, so that part of the misclassification of serum markers is due to failure of liver biopsy itself to accurately differentiate between fibrosis stages.

Still another reason that these markers have performed less well, especially when they have been used more successfully among HCV patients in western countries and among those with more pronounced fibrosis, may involve the nature of African HBV infection.

Despite the high rate of HCC in Senegal, the evolution of these patients with normal ALT is poorly understood and must be considered for specific monitoring in which the LB remains the cornerstone of fibrosis diagnosis.

Compared to adult HBsAg carriers in the Far East and in Western countries, African patients have a lower rate of HBeAg positivity. The pathogenicity of precore mutants is still incompletely understood but probably generally acquired during long-term persistent infection as escape mutants. Precore and core mutations could be associated with more severe liver fibrosis, with discrepancies between the histological, virological, and biochemical stages. [38].

Nevertheless, considering that only two out of eleven patients with an LSM over 13 kPa had contradictory results on biochemical markers, LSM results over 13 kPa might be a reliable measure for initiating treatment. Conversely, an LSM result under 7 kPa should not rule out significant fibrosis, and LB should therefore be performed. The same rule should be applied to patients with LSM values between 7 and 13 kPa.

As potent antivirals such as Tenofovir become more widely available, the only current means of characterizing CHB remains LB in patients with a viral load greater than 3.2 log/mL and normal ALT. Further investigation of surrogate markers adapted to local epidemiological and virological conditions are critically needed.
